# Basal Jawed Vertebrate Phylogenomics Using Transcriptomic Data from Solexa Sequencing

**DOI:** 10.1371/journal.pone.0036256

**Published:** 2012-04-27

**Authors:** Ming Chen, Ming Zou, Lei Yang, Shunping He

**Affiliations:** 1 Key Laboratory of Aquatic Biodiversity and Conservation of Chinese Academy of Sciences, Institute of Hydrobiology, Chinese Academy of Sciences, Wuhan, People's Republic of China; 2 Graduate University of the Chinese Academy of Sciences, Beijing, People's Republic of China; 3 Laboratory of Integrated Biodiversity, Conservation, and Genomics, Department of Biology, Saint Louis University, St. Louis, Missouri, United States of America; Biodiversity Insitute of Ontario - University of Guelph, Canada

## Abstract

The traditionally accepted relationships among basal jawed vertebrates have been challenged by some molecular phylogenetic analyses based on mitochondrial sequences. Those studies split extant gnathostomes into two monophyletic groups: tetrapods and piscine branch, including Chondrichthyes, Actinopterygii and sarcopterygian fishes. Lungfish and bichir are found in a basal position on the piscine branch. Based on transcriptomes of an armored bichir (*Polypterus delhezi*) and an African lungfish (*Protopterus* sp.) we generated, expressed sequences and whole genome sequences available from public databases, we obtained 111 genes to reconstruct the phylogenetic tree of basal jawed vertebrates and estimated their times of divergence. Our phylogenomic study supports the traditional relationship. We found that gnathostomes are divided into Chondrichthyes and the Osteichthyes, both with 100% support values (posterior probabilities and bootstrap values). Chimaeras were found to have a basal position among cartilaginous fishes with a 100% support value. Osteichthyes were divided into Actinopterygii and Sarcopterygii with 100% support value. Lungfish and tetrapods form a monophyletic group with 100% posterior probability. Bichir and two teleost species form a monophyletic group with 100% support value. The previous tree, based on mitochondrial data, was significantly rejected by an approximately unbiased test (AU test, *p* = 0). The time of divergence between lungfish and tetrapods was estimated to be 391.8 Ma and the divergence of bichir from pufferfish and medaka was estimated to be 330.6 Ma. These estimates closely match the fossil record. In conclusion, our phylogenomic study successfully resolved the relationship of basal jawed vertebrates based on transtriptomes, EST and whole genome sequences.

## Introduction

**Figure 1:**
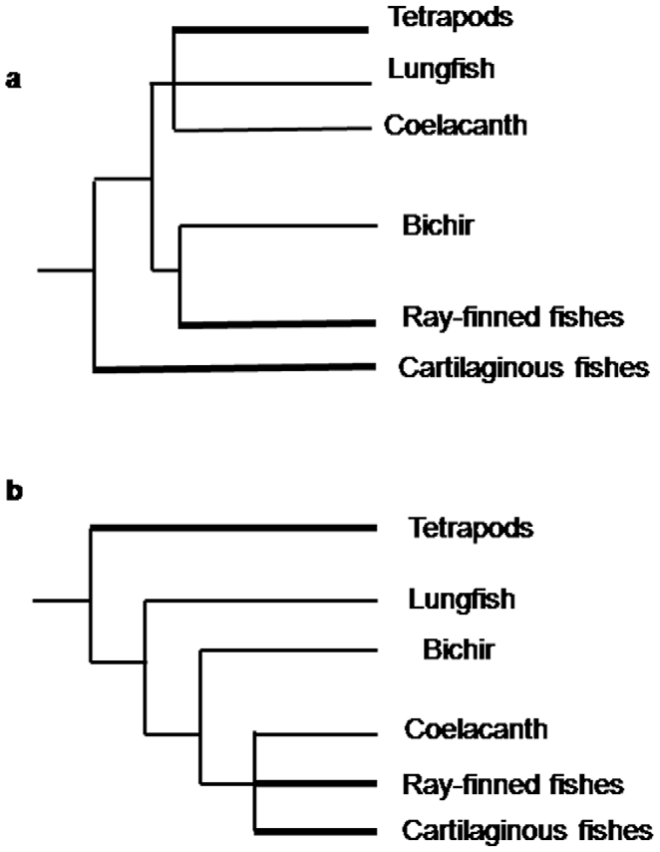
Two hypotheses on the relationships of jawed vertebrates. (A) Traditional view. (B) Mitochondrial tree proposed by Arnason's group [10], [11].

The traditional relationships among jawed vertebrates have been widely accepted by vertebrate zoologists for a long time. Traditionally (Figure 1a), extant jawed vertebrates (gnathostomes) were divided into Chondrichthyes (cartilaginous fishes) and the Osteichthyes (bony vertebrates). The Chondrichthyes have cartilaginous skeletons, separate gill openings (except in chimaeras), and lack endochondral ossification and a lung or swim bladder [1]. Osteichthyes are divided into Actinopterygii (ray-finned fishes) and Sarcopterygii (lobe-finned fishes and tetrapods) based on the attachment of their fins to their bodies. The fins of lobe-finned fishes are connected to the body via a single radial bone, which allows more flexible movement [2], [3]. Tetrapods were thought to have evolved from sarcopterygian fishes based on this and other important characteristics such as the presence of internal nostrils. The Actinopterygii is the other major group of Osteichthyes. It comprises about half of all extant vertebrate species. The four major lineages of basal actinopterygians, Polypteriformes, Acipenseriformes, Lepisosteiformes, and Amiiformes are called “ancient fish”. Generally, Polypteriformes is regarded as the most basal lineage of Actinopterygii [4], [5], [6], [7].

In the 1990s, molecular data was used to study the relationships of extant jawed vertebrates [8], [9]. Around the year 2000, some molecular studies based on mitochondrial sequence data [10], [11] challenged the traditional view. According to these studies, gnathostomes were split into two monophyletic groups: tetrapods and a piscine branch. Lungfish and bichir were placed in a basal position on the piscine branch. This topology (Figure 1b) was supported, or at least not refuted, by some other molecular studies [12], [13].

However, Venkatesh et al. [6] identified 13 derived shared molecular markers (including indels, nuclear introns, and alternatively splicing structure), which support the traditional tree. Dimmick [14] pointed out that their tree was an unrooted tree of basal jawed vertebrates because no outgroup was used. Apart from the position of the bichir, the tree constructed by Venkatesh et al. [6] was equivalent to that constructed by Rasmussen et al. [10], [11], when they were both considered unrooted trees in the comparison. Takezaki et al. [15] showed that the separation of Chondrichthyes (cartilaginous fishes) happened before the divergence of Osteichthyes (bony vertebrates) from the other gnathostomes. However, Takezaki's team used only teleost fishes to represent bony fishes and therefore could not address whether other bony fishes (such as lungfish and bichir) possibly diverged earlier than cartilaginous fishes. Analysis of seven nuclear genes from 14 vertebrate species [16] yielded the traditional vertebrate tree, but as lungfish and tetrapods formed a monophyletic group with only 53% bootstrap support, they proposed that tetrapods might be more closely related to ray-finned fishes than to lungfish. Utilizing Lungfish EST sequences, a recent study [2] claimed to have found significant maximum likelihood support for a traditional gnathostome tree. Gnathostomes were divided into Chondrichthyes and Osteichthyes. Also, lungfish and tetrapods formed a monophyletic group with 100% bootstrap support. However, this study lacked the data on coelacanths, bichir and chimaeras, which may impact the topology. No single molecular data set analyzed to date has included a sufficiently large number of molecular markers and taxa to properly test or confirm this widely accepted hypothesis. The main aim of this study is to do so using a huge number of molecular markers of chimaeras, Neoselachii, bichir, teleosts, lungfish and tetrapods. The availability of whole genome data and many expressed sequences facilitates phylogenetic studies. For cartilaginous fishes, there are many EST sequences of two species of Neoselachii (spiny dogfish, *Squalus acanthias*; little skate, *Leucoraja erinacea*) and a whole genome sequence of a chimaera species (elephant shark, *Callorhinchus milii*). Abundant high quality genomes of tetrapods and teleosts are also available. However, the expressed sequences of basal ray-finned fishes (ancient fishes) and sarcopterygian fishes are not readily available in sufficient quantity. In this study, transcriptomes of an armored bichir (*Polypterus delhezi*) and an African lungfish (*Protopterus* sp.) were sequenced using Solexa sequencing technologies. This is the first use of transcriptomes from Solexa sequencing combined with EST and whole genome sequences to resolve the phylogeny of basal jawed vertebrates.

## Results

The data profile for each species used in this study is shown in Table 1. The transcriptome of the armored bichir contains 24,232 contigs (longer than 100 bp) with a total cumulative length of 3,962,414 bp. There are 22,408 contigs (longer than 100 bp) with a total length of 3,754,165 bp in the transcriptome of the African lungfish. On the basis of these multi-origin expressed sequences (transcriptomes, ESTs, mRNAs, and cDNAs), we obtained 4682 ortholog groups with the help of OrthoSelect [17], [18]. After removing ambiguously aligned blocks and random similarity within multiple sequence alignments, 111 ortholog groups meet our criteria: (a) those found in more than six species; (b) those that contained human single copy genes; and (c) those that included lungfish sequences. The total number of ortholog groups for these 11 taxa and the percentages of missing data from each are shown in Table 1. The supermatrix concatenated from all these 111 ortholog group alignments was 23,262 amino acids long. In final the supermatrix, African lungfish contained 14,739 amino acids. The missing data ranged from 1.8% (human) to 82.4% (elephant shark). The outgroup — sea lamprey contained 92 ortholog groups and missed 22.4% amino acids.

**Table 1:** Data profiles for each species used in the study.

*Taxon name*	*Species name*	*Data type*	*Number of sequences before processing*	*Total length of sequences before processing*	*Number of ortholog groups*	*Percentage of missing amino acids (%)*
human	*Homo sapiens*	cDNA	53564	131391248 bp	111	1.8
mouse	*Mus musculus*	cDNA	40959	99954510 bp	111	2.0
western clawed frog	*Xenopus tropicalis*	cDNA	27711	45111427 bp	104	9.8
African lungfish	*Protopterus* sp.	transtriptome	22408	3754165 bp	111	36.6
armored bichir	*Polypterus delhezi*	transtriptome	24232	3962414 bp	53	73.5
Japanese pufferfish	*Takifugu rubripes*	cDNA	48027	91874005 bp	109	2.6
Japanese medaka	*Oryzias latipes*	cDNA	24662	38371160 bp	105	7.4
elephant shark	*Callorhinchus milii*	Annotated coding sequence	59207	18872940 bp	36	82.4
little skate	*Leucoraja erinacea*	EST and mRNA	15765	10899349 bp	92	24.7
spiny dogfish	*Squalus acanthias*	EST and mRNA	17954	12078559 bp	87	36.1
sea lamprey	*Petromyzon marinus*	EST and mRNA	40963	26813262 bp	92	22.4

**Figure 2:**
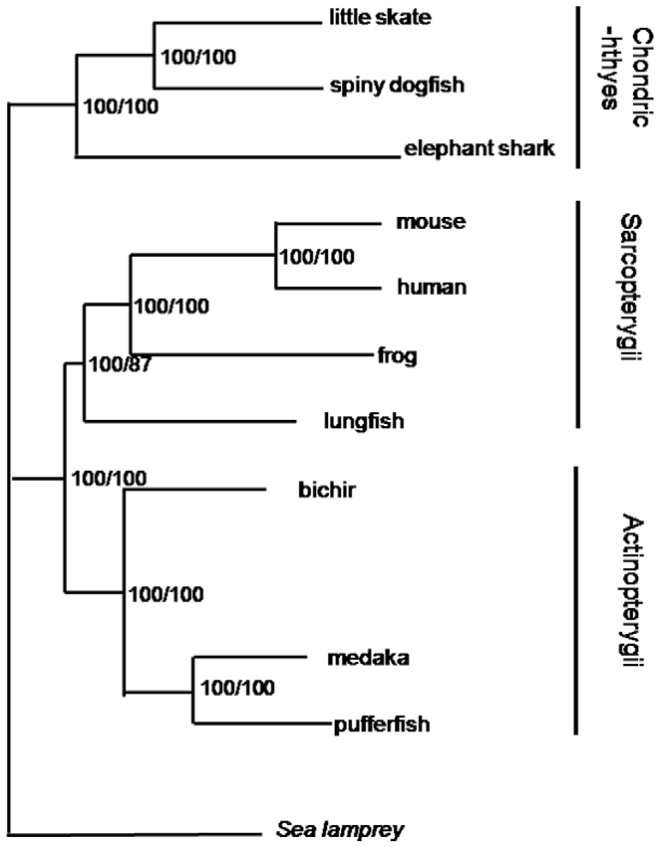
Bayesian tree and Maximum Likelihood tree of basal gnathostomes. Posterior probabilities and bootstrap values are indicated before and after a slant, respectively.

According to the BI and ML trees of basal gnathostome relationships (Figure 2) inferred from the 111 genes, the gnathostomes are divided into Chondrichthyes and Osteichthyes. Both Chondrichthyes and Osteichthyes are recovered as monophyletic with 100% posterior probabilities and bootstrap values. Rasmussen and Arnason [10], [11] found cartilaginous fishes in a terminal position in their trees. However, their topology (Figure 1b) was significantly rejected by AU test (*p* = 0).

For Chondrichthyes, chimaeras may be considered to have derived from placoderms independently from other cartilaginous fishes as pointed out by some paleontologists [16], [19] Figure 2 shows that chimaeras have a basal position among cartilaginous fishes with 100% posterior probabilities and bootstrap values.

For Sarcopterygii, lungfish and tetrapods form a monophyletic group with 100% posterior probabilities (Figure 2). Our results support that tetrapods originated from sarcopterygian fishes. But because we do not have the data of coelacanth, the relationships of lungfish, coelacanth and tetrapods cannot be resolved by this study.

For Actinopterygii, bichir, Japanese pufferfish and Japanese medaka form a monophyletic group with 100% support values. Because bichir share many characteristics with both lobe-finned fishes and ray-finned fishes [1], [6] the phylogenetic position of bichir has been subject to much debate. Most studies currently place bichir in a basal position in ray-finned fishes [4], [5], [6], [7]. However, Arnason's group proposed that bichir are basal to all other piscine species. This topology was significantly rejected (AU test, *p* = 0).

**Figure 3:**
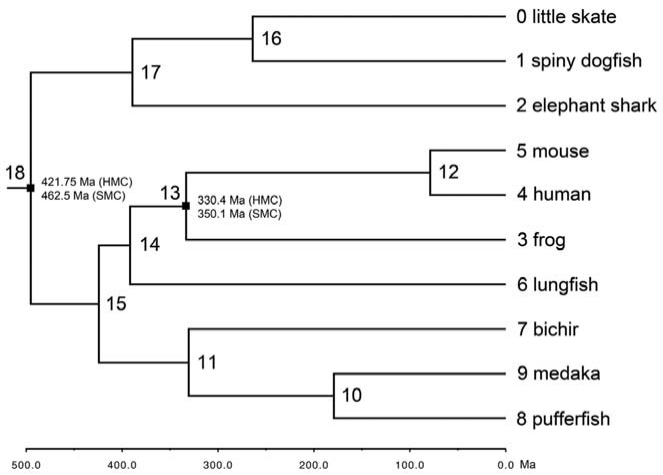
Divergence time estimate of basal jawed vertebrates. The assigned node numbers are showed (see also Table 2). The two nodes (18 & 13) used for calibration are indicated. HMC: hard minimum constraint; SMC: soft maximum constraint.

The different assigned nodes of gnathostomes, including two fossil calibration points [20], is shown in Figure 3: Chondrichthyes-Osteichthyes (18), 421.8–462.5 Ma [21], [22], [23], [24]; frog -human, mouse (13), 330.4–350.1 Ma [25] Table 2 gives the mean divergence time values and the 95% highest posterior density (HPD) interval for the nodes in Figure 3. For example, the divergence time of Chondrichthyes and Osteichthyes was dated to the Cambrian period (495.2 Ma); the lungfish-tetrapods divergence was estimated to be 391.8 Ma (Devonian). We estimate that elasmobranchs and chimaeras also diverged during the Devonian (389.3 Ma).

**Table 2:** Divergence times in Ma with 95% highest posterior density (HPD).

Node	BEAST (Ma)	95% HPDa(Ma)^a^
10	179.2	129.9–226.1
11	330.6	276.9–385.8
12	78.8	52.5–110.1
13	333.3	330.5–342.1
14	391.8	363.4–426.1
15	424.2	386.1–465.5
16	263.8	187.8–329.4
17	389.3	310.1–459.2
18	495.2	424.1–552.5

a The lower bound and higher bound of the 95% HPD interval. The 95% HPD is shortest interval that contains 95% of the sampled values.

## Discussion

Our phylogenetic analysis supported the traditional view of relationships among basal jawed vertebrates (Figure 1a). The relationships based on mitochondrial DNA of Arnason's group (Figure 1b) were rejected by AU test (*p* = 0). This “odd topology” may be due to noise (saturation) in the molecular data [26] In addition, the evolutionary rate of mitochondrial sequences of tetrapods is much faster than those of fishes [27], [28]. These evolutionary features of mitochondrial sequences can cause Long Branch Attraction artifacts [16], [29].

The lungfish–tetrapods divergence was estimated at 391.8 Ma when left unconstrained. The 395-My-old fossil *Kenichthys* [30] represents the oldest member of the Tetrapodomorpha, which is a clade of sarcopterygians with tetrapod features. The molecular estimate was close to the date given by the fossil record. The estimated time of divergence of bichir and medaka from pufferfish was 330.6 Ma, which also matches the fossil record well (392.0 Ma) [31].

The divergence time of Actinopterygii and Sarcopterygii was dated to the Silurian period (424.2 Ma). The lungfish-tetrapods divergence was estimated at 391.8 Ma (Devonian). We determined that elasmobranchs and chimaeras diverged during the Devonian (389.3 Ma). These observations suggest that the early divergences among basal gnathostomes took place within a narrow temporal window.

Expressed sequences are a powerful tool for producing protein-coding sequences for phylogenetic studies [32], [33], [34]. For some species, like African lungfish, which have a very large genome, genome sequencing projects may be unrealistic with current DNA sequencing technology. However, next-generation RNA-Seq may solve this problem because it makes it easy to obtain the transcriptomes of these species. Next-generation RNA-Seq has also been found to obtain more sequences than previous EST and cDNA sequencing methods.

Our phylogenomic study based on transcriptomes from Solexa sequencing combined with other ESTs and whole genome sequences successfully resolved major phylogenetic problems of basal gnathostomes. However, our phylogenetic analysis does not completely resolve these relationships because of the lack sequences from the coelacanth. Further analyses should include those data. The next generation RNA-Seq technology can provide more abundant and high quality transcriptomes from these species, which may further resolve these problems.

## Materials and Methods

### Data collection and processing

Transcriptomes of an African lungfish (*Protopterus* sp.) and an armored bichir (*Polypterus delhezi*) were generated using Solexa sequencing. Total RNA was extracted from each species from pooled organs using Trizol (Invitrogen, Carlsbad, CA, U.S.) according to the manufacturer's instructions. Poly (A+) RNA isolation, cDNA synthesis, preparation, and sequencing (on an Illumina Genome Analyzer) were performed at the Beijing Genomics Institute. The assembly procedure was conducted as described by Li et al. [35]. Short reads were assembled to construct contigs using SOAPdenovo software [36].

Expressed sequences (ESTs and mRNAs) of sea lamprey (*Petromyzon marinus*), spiny dogfish (*Squalus acanthias*), and little skate (*Leucoraja erinacea*) were downloaded from the National Center for Biotechnology Information (www.ncbi.nlm.nih.gov). Various contaminants, low quality and low-complexity sequences from these data were screened and trimmed using SeqClean (http://compbio.dfci.harvard.edu/tgi/software) with NCBI's UniVec serving as a screening file. Complementary DNA sequences of two model fish species: Japanese pufferfish (*Takifugu rubripes*) and Japanese medaka (*Oryzias latipes*), and three tetrapod species: human (*Homo sapiens*), mouse (*Mus musculus*), western clawed frog (*Xenopus tropicalis*) were retrieved from Ensembl (http://www.ensembl.org/, RELEASE50).

The whole genome sequence of the elephant shark (*Callorhinchus milii*) was downloaded from http://esharkgenome.imcb.a-star.edu.sg/. Coding regions were annotated according to the annotated protein datasets of eight chordate species (*Ciona intestinalis*, *Takifugu rubripes*, *Gasterosteus aculeatus*, *Oryzias latipes*, *Danio rerio*, *Xenopus tropicalis*, *Gallus gallus*, *Homo sapiens*) obtained from Ensembl. A TBLASTN [37] search was performed using these protein sequences as queries against the whole genome sequences of elephant shark to identify homologous regions. Genewise [38] was used to define the gene structure of these homologous regions. A Perl script was used to distill coding sequences from the Genewise results. According to Genewise results, the sequences whose open reading frames were disrupted (by frameshift mutations or premature stop codons) were defined as pseudogenes and were removed from the data.

### Sequence selection and alignment

**Figure 4:**
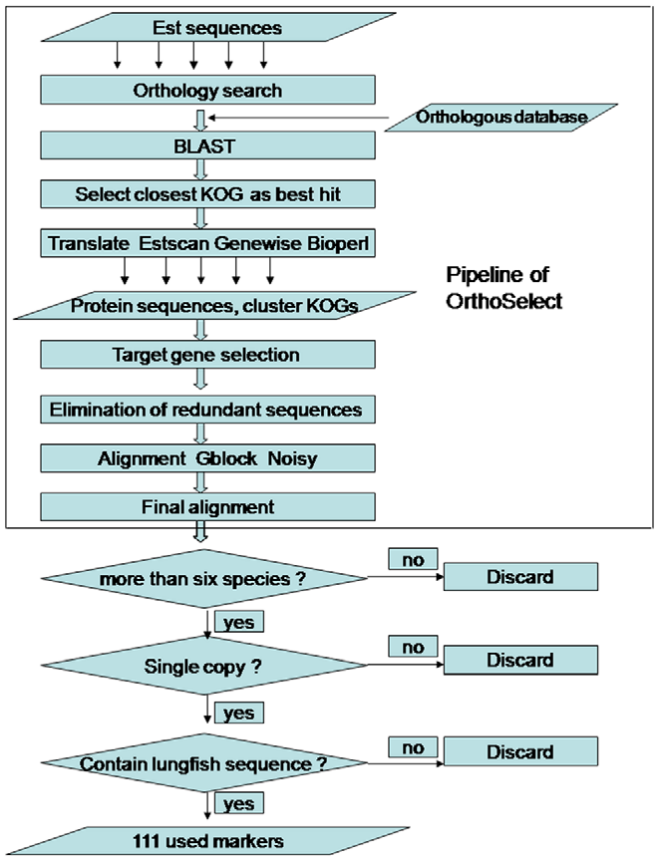
Pipeline to select gene markers for final tree construction and divergence time estimation.

Orthologs are commonly defined as genes that have diverged after a speciation event [39]. Identifying orthologs correctly is key to reconstructing phylogenetic trees. Ortholog assignment was achieved using the OrthoSelect method [18]. KOG ortholog groups included many ortholog groups, and each group consisted of many eukaryotic protein sequences. For each ortholog group, using BLASTX, each EST sequence that reached the threshold was associated to a protein, and if there was more than 1 sequence, we selected the best one (lowest e-value) [40], [41]. Using different translation tools (ESTScan, GeneWise, and a standard six-frame translation using BioPerl) [42], we translated each EST sequence to protein sequence, and aligned to its associated protein sequence using bl2seq [43], to find the most probable translation strategies. Multiple divergent copies of the same gene and different levels of stringency during EST assembly sometimes led to situations in which KOGs contained more than one sequence per species. To eliminate redundant sequences, one sequence from each organism was selected to represent the most probable ortholog to each other in accordance with Schreiber et al.'s [18] strategy based on matching positions normalized by length in pairwise comparisons using MUSCLE [44]. Then, Gblocks [45], [46] and Aliscore [47] were used to remove ambiguously aligned blocks and random similarity within multiple sequence alignments, respectively. We chose ortholog groups for further analysis using the following three criteria: (a) those found in more than six species; (b) those containing human single copy genes; and (c) those that included lungfish sequences. The pipeline for the selection of genetic markers is shown in Figure 4.

### Phylogenetic analysis

We concatenated alignments of these ortholog groups into a single alignment, and then the concatenated protein matrix was subjected to Bayesian inference (BI) and Maximum likelihood (ML) analyses. Bayesian inference was performed using the MPI version of MrBayes 3.1.2 [48], [49], in which Markov chain Monte Carlo (MCMC) calculations were spread across multiple CPUs and run on parallel computing architectures. The analysis was initiated from a random starting tree. Two runs with 32 chains of MCMC iterations were performed for 1 million generations (sampling trees every 100 generations), and the first 2500 trees (250,000 generations) were discarded as burn-ins. The average standard deviation of split frequencies (ASDSF) of the MCMC runs was used as convergence diagnostics. The 50% majority-rule consensus tree was determined to calculate the posterior probabilities for each node. The parallel version of RAxML 7.2.6 [50], [51] was used for constructing maximum likelihood (ML) trees. Prottest [52], [53], [54] was used to obtain the best model for each orthologous gene. Sea lamprey was used as outgroup to root the tree. The datasets were partitioned to allow RAxML to assign different parameters for each gene. One hundred replicates for rapid bootstrap analyses [55] were also performed using RAxML, and a 50% majority-rule consensus was calculated to determine the support values for each node. Tests of alternative phylogenetic hypotheses were implemented in CONSEL [56].

### Estimation of time of divergence

Divergence time was estimated using BEAST v.1.6.2 [20] via the CIPRES Science Gateway v.3.1 [57]. BEAUti v.1.6.1 [20] was used to generate the XML file for BEAST. The following model was employed: Blosum62+I+G (4 categories). We selected “Relaxed Clock: Uncorrelated Lognormal” as clock model and “Speciation: Yule Process” as tree prior. The best Maximum Likelihood tree obtained from previous analysis was used as starting tree. A lognormal prior distribution was adopted because it fixed the minimum age (the “hard minimum constraint”) of a calibrated node and allowed the maximum age (the “soft maximum constraint”) to be sampled following a lognormal distribution [58] Two nodes (18 & 13), each with a hard minimum constraint and a soft maximum constraint, were used for calibration (Figure 3). The hard minimum constraint and soft maximum constraint of node 18 were set as 421.75 Ma and 462.5 Ma, respectively. The oldest phylogenetically secure record of the divergence of crown gnathostomes is established on the basis of *Andreolepis hedei* [21], [22], [23], [24]. This is at least a stem-Osteichthyan, if not a stem-Actinopterygian. The oldest record of *A. hedei* is established on the basis of a graphic correlation composite standard, at 421.75 Ma. The soft maximum constraint can be established on the basis of the oldest phylogenetically secure stem-gnathostome, *Sacabambaspis janvieri* [24], [59], dated at 462.5 Ma. For node 13, we chose 330.4 Ma as the hard minimum constraint and 350.1 Ma as the soft maximum constraint [59]. The hard minimum constraint is based on the oldest reptiliomorph fossil *Lethiscus stocki* [59]. The soft maximum constraint is established on the basis of the oldest whatcheeriid fossils *Whatcheeria* and *Pederpes* [59]. In BEAUti, the mean and standard deviation for the prior distribution of node 18 were set as 2.062 and 1.0, respectively. For node 13, the above two parameters were set as 1.335 and 1.0, respectively. These parameters have been manually adjusted so that 95% of the prior distribution lies between the hard minimum constraint and soft maximum constraint. All other parameters in BEAUti were left at default. Markov Chain Monte Carlo (MCMC) analyses were run for 10,000,000 generations (parameters sampled every 1000 generations). Tracer v.1.5 [20] was used to summarize BEAST output, discarding one million generations as burn-in. One maximum clade credibility tree was created using TreeAnnotator v.1.7.0 [20] with a 0.5 posterior probability limit, discarding 1000 trees as burn-in. The 95% Highest Posterior Density (HPD) limits of the node heights were summarized. FigTree v.1.3.1 (http://tree.bio.ed.ac.uk/software/figtree) was then used to visualize the results.
